# Direct-coupled micro-magnetometer with Y-Ba-Cu-O nano-slit SQUID fabricated with a focused helium ion beam

**DOI:** 10.1063/1.5048776

**Published:** 2018-10-15

**Authors:** Ethan Y. Cho, Hao Li, Jay C. LeFebvre, Yuchao W. Zhou, R. C. Dynes, Shane A. Cybart

**Affiliations:** 1 Department of Mechanical Engineering, University of California, Riverside, 900 University Ave., Riverside, California 92521, USA; 2 Department of Physics, University of California, Riverside, 900 University Ave., Riverside, California 92521, USA; 3 Department of Physics, University of California, San Diego, 9500 Gilman Dr., La Jolla, California 92093, USA; 4 Materials Science and Engineering, University of California, Riverside, 900 University Ave., Riverside, California 92521, USA

## Abstract

Direct write patterning of high-transition temperature (high-*T*_C_) superconducting oxide thin films with a focused helium ion beam is a formidable approach for the scaling of high-*T*_C_ circuit feature sizes down to the nanoscale. In this letter, we report using this technique to create a sensitive micro superconducting quantum interference device (SQUID) magnetometer with a sensing area of about 100 × 100 *μ*m^2^. The device is fabricated from a single 35-nm thick YBa_2_Cu_3_O_7−__*δ*_ film. A flux concentrating pick-up loop is directly coupled to a 10 nm × 20 *μ*m nano-slit SQUID. The SQUID is defined entirely by helium ion irradiation from a gas field ion source. The irradiation converts the superconductor to an insulator, and no material is milled away or etched. In this manner, a very narrow non-superconducting nano-slit is created entirely within the plane of the film. The narrow slit dimension allows for maximization of the coupling to the field concentrator. Electrical measurements reveal a large 0.35 mV modulation with a magnetic field. We measure a white noise level of 2 *μ*Φ_0_/Hz^1∕2^. The field noise of the magnetometer is 4 pT/Hz^1∕2^ at 4.2 K.

Superconducting quantum interference devices (SQUIDs) are used in an amazingly diverse range of applications requiring sensitive detection of magnetic flux.^1,2^ Commercial SQUID systems are currently used in medicine,^3,4^ materials science,^5^ geology,^6,7^ and cosmology.^8^ Experimental SQUIDs are at the forefront in many areas of developmental high-performance low noise electronics. Recent work has shown that scaling SQUIDs to very small dimensions is a viable approach to reduce flux noise. Nano-SQUID devices have been demonstrated with very low values of flux noise below 1 *μ*Φ_0_/Hz^1∕2^.^9,10^ Unfortunately, the small effective area results in a very low field sensitivity of the order of nT/Hz^1∕2^. To achieve high sensitivity, flux must be concentrated into the SQUID.

Most commercial SQUID magnetometers are fabricated from low temperature superconductors (LTS) and are based on a design developed over three decades ago by Ketchen and Jaycox.^11^ The Ketchen-Jaycox (KJ) magnetometer consists of two essential components. The first is the SQUID, which consists of two Josephson junctions connected in parallel by a superconducting loop of the order of tens of micrometers on a side. The second component is a superconducting multi-turn transformer that concentrates flux from a large area primary coil into a multi-turn secondary coil coupled to the small loop of the SQUID. The measure of concentration is measured in units of magnetic field per flux quantum (nT/Φ_0_). The product of the flux concentration and the magnetic flux noise ($S_{\Phi }^{1/2}$), usually quoted in units of *μ*Φ_0_/Hz^1∕2^, is the magnetic sensitivity of the magnetometer in the familiar field noise ($S_{B}^{1/2}$) units of pT/Hz^1∕2^. The KJ magnetometer requires multiple superconducting layers separated by insulators with vias and crossovers to connect the multi-turn transformer back on to itself. Furthermore, LTS versions often utilize a coil of superconducting niobium wire for the primary side of the transformer.

While production of KJ magnetometers is a straight-forward process in LTS materials, this is not the case for high-transition temperature (high-*T*_C_) materials. Fabrication of multi-layers and vias is especially challenging because the layers must be epitaxially grown at high temperatures and lithographically patterned and etched in between each layer of growth. The growth process is also complicated by the difficulty in growing defect free single crystals of the complex unit cell structures and chemical stoichiometry. Furthermore, the high-*T*_C_ cuprates are brittle ceramic materials which cannot be easily made into wires. Although, high-quality high-*T*_C_ coated tapes have been developed for power transmission, they are difficult to use in magnetometer applications because they have large bend radii and metallic substrates that contribute Nyquist noise to the system. Despite these challenges, there have been demonstrations of high-*T*_C_ multi-layer KJ style magnetometers^12–14^ but the difficult fabrication process is time consuming with very low yields. The field noise levels also tend to be very high 50 fT/Hz^1∕2^ at 10 Hz.^15^

Faley *et al.*^16^ significantly improved the field noise of high-*T*_C_ magnetometers using an alternative to the KJ magnetometer. In this work, a multi-turn flux transformer fabricated on a separate chip is bonded face-to-face with a single layer SQUID in a flip-chip configuration. Several individual transformers and SQUIDs are made separately and those with the lowest noise are selected for the combined magnetometer. In this manner, devices with extremely low noise, approaching that of the best commercial LTS SQUID magnetometers ∼10 fT/Hz^1∕2^ have been demonstrated.^1^ While commercialization and large-scale fabrication of sensors using this approach is not currently economically feasible, these devices highlight the remarkably low levels of noise that are obtainable in high-*T*_C_ devices. While simpler in construction in comparison to KJ integrated magnetometers, the flip-chip transformer still requires multi-layer deposition and patterning to connect the multi-turn transformer back onto itself. Due to the complexity and high costs of high-*T*_C_ multi-layer technology, a different approach that features both a concentrator and SQUID in a single layer of high-*T*_C_ is very desirable.

The most successful single layer approach is referred to as a direct-coupled magnetometer (DCM), where a superconducting pick-up loop is connected to a SQUID by a shared electrode.^17,18^ This form of coupling is direct, opposed to inductive like in most other configurations. The magnetic flux treading the pick-up loop induces a superconducting current which also circulates through the SQUID. The high impedance of the Josephson junctions ensures that the induced current flows through the inductance of the SQUID, hereby generating a flux that is sensed by the SQUID. The effective area of the DCM is *A*_s_ + *αA*_p_*L*_s_/*L*_p_, where *A*_p_, *L*_p_ and *A*_s_, *L*_s_ are the area and inductance of the pick-up loop and the SQUID, respectively, and *α* is a constant related to the shared electrode. The DCM effective area is ∼*αA*_p_*L*_s_/*A*_s_*L*_p_ times larger than the bare SQUID. This design was experimentally implemented with much success, demonstrating field sensitivity as low as 30 fT/Hz^1∕2^ for a 2 cm × 2 cm sensor.^19^ However, this value remains an order of magnitude greater than that of similar sized flip-chip magnetometers. To minimize noise, the optimal effective area is $A_{s}+\alpha A_{p}L_{s}/L_{p}(\sqrt{L_{p}/L_{s}}/2)$, which is $∼\sqrt{L_{p}/L_{s}}/2$ times larger than the direct coupled design.^14^ In other words, the performance of the DCM can be improved by matching *L*_p_ to *L*_s_ while maximizing the shared current path by the two loops. The most successful direct coupled geometries achieve this by using a long narrow slit. Additionally, the field sensitivity can be improved by reducing the intrinsic flux noise from the SQUID. For small SQUIDs, the flux noise can be estimated by $16k_{B}TL_{s}^{2}/R$, where *T* is the ambient temperature and *R* is the resistance of the SQUID.^20^ A smaller SQUID loop decreases *L*, whereas smaller junctions increase *R*.

Recent progress in helium ion beam direct write patterning of high-*T*_C_ superconductors provides an approach for controlling these variables.^21,22^ This technique can reliably produce nanoscale structures in YBa_2_Cu_3_O_7−__*δ*_ (YBCO), allowing unprecedented control of *L* and *R* in high-*T*_C_ devices.^23^ The values of *R* are typically 1 to 2 orders of magnitude higher than prior-art ion damage junctions.^24,25^ Additionally, the helium ion patterning technique provides a means to create sub-10 nm structures in YBCO several orders of magnitude smaller than photolithography. This allows for the slit width to be reduced to the nanoscale so that the shared current path between the concentrator and SQUID can be made as long as the inductance permits.

In this letter, we report the use of helium ion beam direct patterning to develop a nano-slit SQUID in a direct coupled configuration for a total magnetometer size of 100 × 100 *μ*m^2^ for the sensing element of a Tristan Technologies inverted SQUID microscope. The usage of YBCO opposed to the conventional niobium SQUID allows for the development of a sensor that can operate at the optimal working temperature of the system ∼10 K. We utilize the very low flux noise of a small area nano-slit SQUID, which is directly coupled to a pick-up loop for increased sensitivity.

To begin the fabrication process, a 35-nm thick YBa_2_Cu_3_O_7−__*δ*_ (YBCO) thin film was deposited onto cerium oxide buffered sapphire by Ceraco Ceramic Coating GmbH using reactive coevaporation.^26^ Subsequently, a 250-nm layer of gold was thermally evaporated over the YBCO *in-situ* for electrical contacts. Large scale features, shown in Fig. 1(a), such as the pick-up loop, control line, and electrical contacts were patterned into both the gold and YBCO layers using photolithography followed by argon ion milling. In a second photolithography step, the gold covering the SQUID and flux concentrator sections of the design was removed by a KI-I^+^ gold etchant. Samples were then loaded onto a Zeiss Orion Plus microscope for helium ion irradiation. Figure 1(b) shows a photograph of a 30 × 30 *μ*m^2^ area of the device that contains the nano-slit SQUID. The finely focused helium beam was scanned across the region [Fig. 1(b) dashed white line] delivering a dose of 6 × 10^17^ ions/cm^2^ to convert a narrow 0.2 *μ*m^2^ (10 nm × 20 *μ*m) slit from a superconductor to an insulator, forming the loop of the SQUID. Two Josephson junctions were then written with a dose of 8 × 10^16^ ions/cm^2^ on the left side of the slit [Fig. 1(b) solid red lines] that were trimmed to 2 *μ*m by subsequent irradiation of two rectangular insulators. While simulations using Silvaco estimate the insulating slit of the SQUID to be only 10 nm,^21^ we assume that the magnetic field penetrates a distance of the two dimensional penetration depth $\lambda_{⊥}=\lambda_{L}^{2}/t∼1.1\;\;\mu m$, where *λ_L_* is the London penetration depth (∼200 nm) and *t* is the thickness of the film (35 nm). The penetration results in a larger effective area of 2*λ*_⊥_ × 20 *μ*m.

**FIG. 1.**
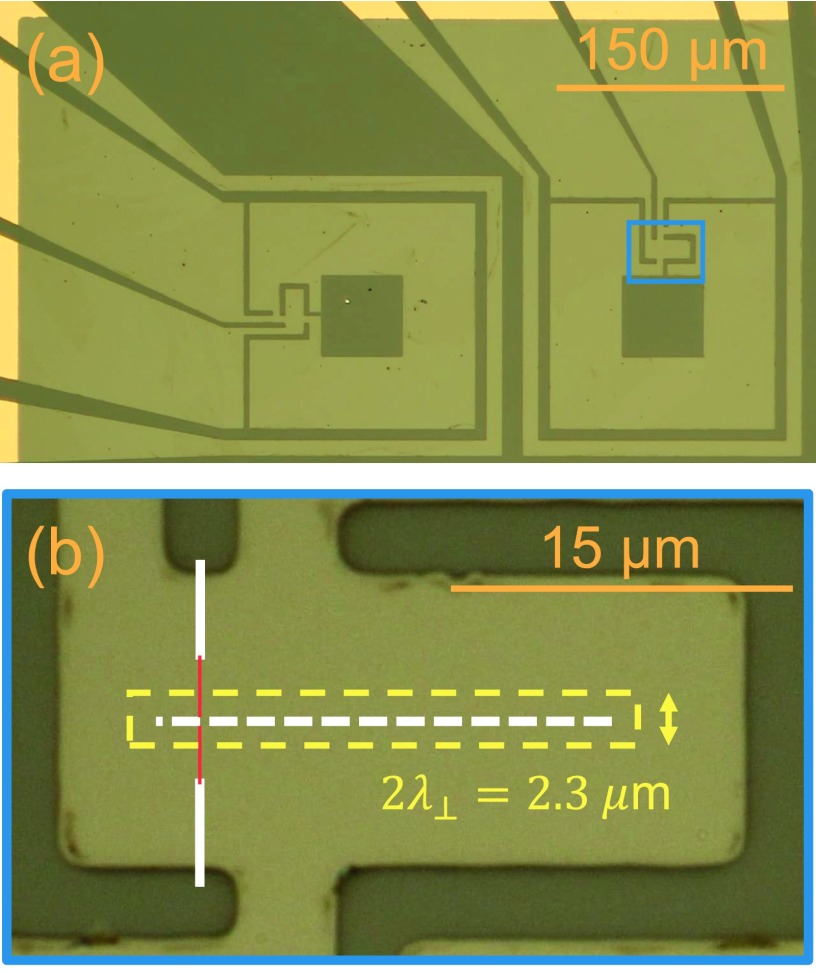
(a) Photograph of two YBCO nano-slit SQUID direct coupled magnetometers with single turn control lines. The square washer like loop is connected in parallel with the nano-slit SQUID for flux concentration. The nano-slit SQUID of the right-most device is highlighted and enlarged below. (b) A narrow slit (white dashed line) is irradiated with the focused ion beam converting the YBCO from a superconductor to an insulator. Two 2 *μ*m wide Josephson junctions are subsequently irradiated (solid red lines) along with two insulators (white rectangles). Although the actual SQUID loop is only 10 nm wide by 20 *μ*m, line flux can penetrate up to the two dimensional penetration depth (dotted yellow rectangle).

We remark that YBCO is highly sensitive to ion irradiation and the doses used in this process are at least several orders of magnitude lower than that required to mill or remove the material. As a consequence, the patterned structures are not visible after writing. However, electrical transport data to be presented below strongly corroborate that the lithographic and actual physical dimensions are commensurate with one another.

For electrical testing the nano-slit SQUID micro magnetometer was mounted on a liquid helium dip probe equipped with a *μ*-metal shield and cooled to 4.2 K. The current-voltage characteristics (*I-V*) were measured and are shown in Fig. 2 with characteristics of the resistively shunted junction model. The junctions are well-described by the resistively shunted junction model and feature insulating Josephson barriers with no excess current like those in our prior work.^21^ The critical current (*I*_C_) and voltage state resistance (*R*) from *I-V* were 65 *μ*A and 11 Ω, respectively.

**FIG. 2.**
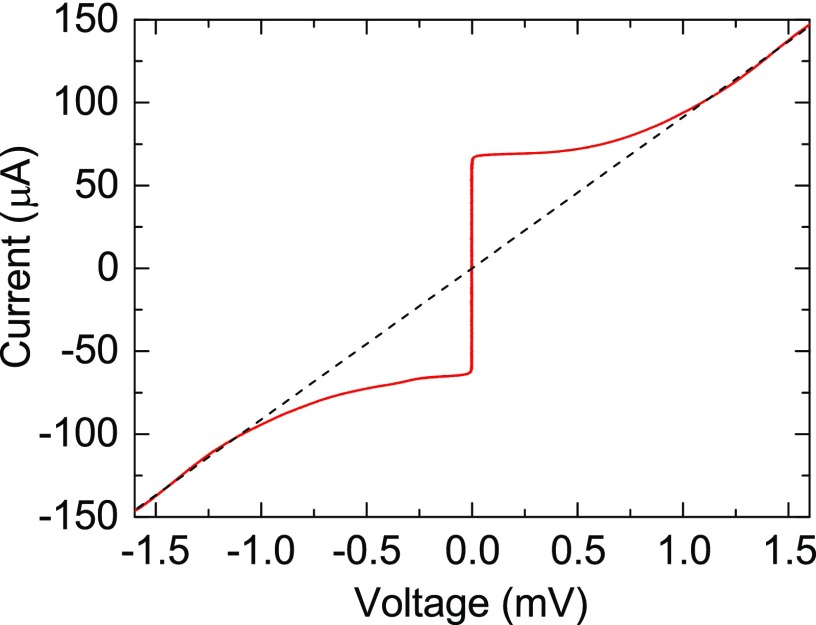
Current-voltage characteristic for the direct coupled nano-slit SQUID magnetometer measured at 4.2 K. The *I*_C_ of the SQUID is 65 *μ*A, and the resistance of 11 Ω is shown with a dashed line.

To investigate the magnetic field properties, the SQUID was biased with a static current of 68 *μ*A and the voltage was measured as a function of magnetic field applied from an external solenoid. Figure 3(a) shows data for a large field range of ±100 *μ*T. In this range, we can observe the high frequency oscillation from quantum interference between the two Josephson junctions with a large range amplitude modulation from the Fraunhofer diffraction related to the area of the junctions. The Fraunhofer pattern decreases to be around the value of *I*_C_ *R* near 100 *μ*T, which corresponds to an estimated Josephson junction width of 6 *μ*m using the calculation reported by Rosenthal *et al.*^27^ This is an interesting result because the junction width is trimmed to 2 *μ*m (from 6 *μ*m) with a high dose of irradiation turning the YBCO insulating. We believe the discrepancy is due to an additional focusing contribution focused into the junctions from the electrodes. Another interesting feature worth noting in the large range voltage-magnetic field characteristic is the presence of a 50 *μ*T beat in the amplitude. We attribute this oscillation to be a response of the SQUID to its own individual area aside from that of the concentrator. Assuming this area to be equal to the length of the [(20 *μ*m + 2*λ*_⊥_) × 2*λ*_⊥_], we obtain a value for *λ*_⊥_ = 1.16 *μ*m which is consistent with our prior calculation.

**FIG. 3.**
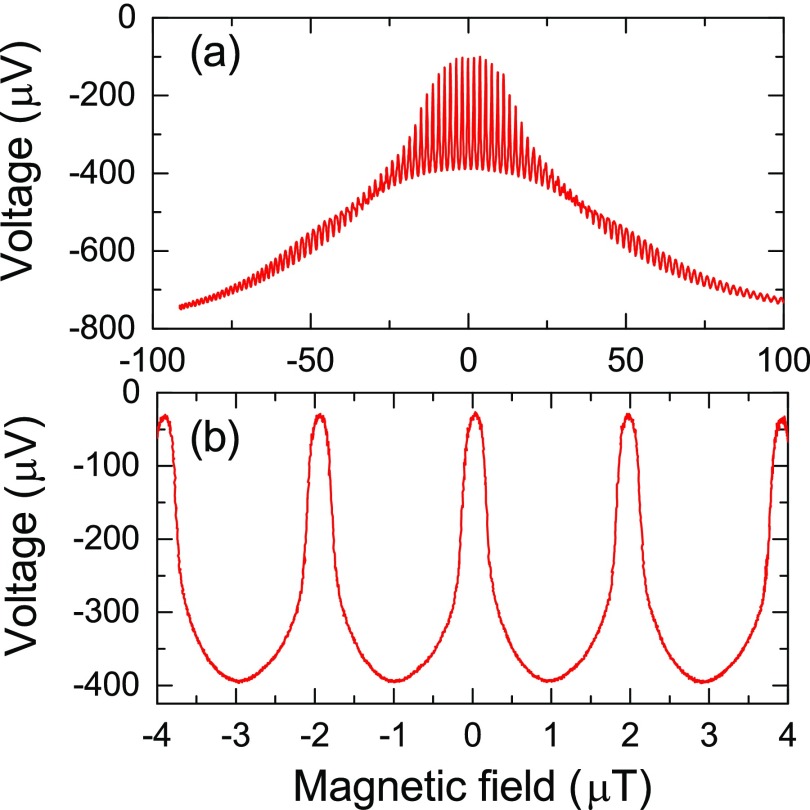
(a) Voltage as a function of the magnetic field for the direct coupled nano-slit SQUID magnetometer measured with a DC bias current of 68 *μ*A. Over a hundred oscillations are visible from quantum interference between the junctions with amplitude modulated from Fraunhofer diffraction from flux threading the area of the junctions. A 50 *μ*T beating of the amplitude is also observed which we attribute to the effective area of the nano-slit SQUID itself. (b) Is a zoomed in view of the oscillations near zero field exhibiting a nearly ideal interference pattern with a relatively large 0.35 mV amplitude, 2 *μ*T period, and a maximum *V*/Φ ∼ 5300 V/Φ_0_.

The interference oscillations near zero field are shown in Fig. 3(b), illustrating a large modulation amplitude of 350 *μ*V. This value corresponds to $\frac{1}{2}I_{C}R$ from Fig. 2 which strongly suggests an inductive parameter *β*_L_ = 2*I*_C_*L*/Φ_0_ ∼ 1 based on the work of Tesche and Clarke. This implies an inductance of 32 pH. The very sharp near ideal DC SQUID interference has a maximum *dV*/*dB* value of 2800 *μ*V/*μ*T with a period of 2 *μ*T/Φ_0_ corresponding to a *dV*/*d*Φ of 5300 *μ*V/Φ_0_.

Noise was measured by biasing the SQUID with a static current of 68 *μ*A, and 0.25 *μ*T field while measuring the amplified voltage with a spectrum analyzer (Fig. 4). The white flux noise was determined to be 2 *μ*Φ_0_/Hz^1∕2^. The field sensitivity of the magnetometer determined by multiplying the field periodicity and noise is 4 pT/Hz^1∕2^.

**FIG. 4.**
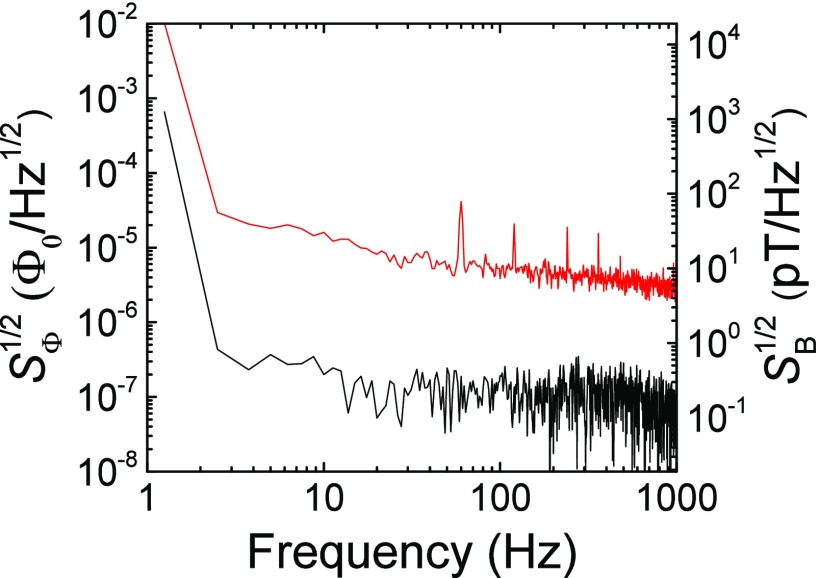
Noise spectrum for the direct coupled nano-slit SQUID magnetometer measured at a bias current of 68 *μ*A without flux-locked loop electronics and bias reversal (red). $S_{\Phi }^{1/2}$ and $S_{B}^{1/2}$ (red spectrum) were determined using *V*-Φ from the data in Fig. 3. The black spectrum is the baseline of the preamplifier used for the measurement.

This work demonstrates the versatility of direct write helium ion patterning of high-*T*_C_ films by material modification. The power of this approach is that no material is milled or removed and by interaction with the beam at moderate doses, the superconductivity is destroyed on the nm scale to create insulators and electrical pathways entirely within the plane of the film. A limit imposed by the nature of this process is that subsequent high temperature processing cannot be performed without adversely affecting the junction. For multi-layer structures, all of the films would have to be deposited and etched before ion beam writing and underlying layers need to be deeper than the range of the ions (∼100 nm) to prevent damage.

While this sensor was designed for a low temperature application, the same approach can be utilized at higher temperature. We believe the ability to create both junctions as well as SQUID loops at this scale enables an alternative approach for fabrication of high-*T*_C_ devices requiring micron sized sensors. This approach could significantly improve the sensitivity of SQUID arrays for high frequency communication components^28–30^ by allowing more flux concentration. Scaling to these small dimensions could also enable new applications in digital electronics such as adiabatic quantum flux parametron.^31^ The ability to control the important device parameters such as resistance, critical current, and inductance will have a significant impact on future high-*T*_C_ devices.
